# Fluorescence-assisted sequential insertion of transgenes (FASIT): an approach for increasing specific productivity in mammalian cells

**DOI:** 10.1038/s41598-020-69709-1

**Published:** 2020-07-30

**Authors:** Felipe E. Bravo, Natalie C. Parra, Frank Camacho, Jannel Acosta, Alaín González, Jorge R. Toledo, Oliberto Sanchez

**Affiliations:** 1 Laboratorio de Biofármacos Recombinantes, Departamento de Farmacología, Facultad de Ciencias Biológicas, Universidad de Concepción, P.O. Box 160C, Concepcion, Chile; 2 Laboratorio de Biotecnología y Biofármacos, Departamento de Fisiopatología, Facultad de Ciencias Biológicas, Universidad de Concepción, Concepcion, Chile; 3 Centro de Biotecnología y Biomedicina Spa., Vilumanque 186, Concepcion, Chile

**Keywords:** Expression systems, Biological techniques, Biotechnology

## Abstract

Currently, the generation of cell lines for the production of recombinant proteins has the limitation of unstable gene expression due to the repeat-induced gene silencing or the loss of transgene copies resulting from recombination events. In this work, we developed a new strategy based on the sequential insertion of transgenes for generating stable clones producing high levels of a chimeric human follicle-stimulating hormone (hscFSH). Gene insertion was done by transducing HEK-293 cells with a lentiviral vector containing a bicistronic transcriptional unit for expressing hscFSH and GFP genes. Clone selection was performed by flow cytometry coupled to cell sorting, and the GFP gene was further removed by CRE-mediated site-specific recombination. High-producing clones of hscFSH were obtained after three rounds of lentiviral transduction. Expression levels increased in a step-wise manner from 7 to 23 pg/cell/day, with a relatively constant rate of 7 pg/cell/day in each round of transduction. The GFP gene was successfully removed from the cell genome without disturbing the hscFSH gene expression. Clones generated using this approach showed stable expression levels for more than two years. This is the first report describing the sequential insertion of transgenes as an alternative for increasing the expression levels of transformed cell lines. The methodology described here could notably impact on biotechnological industry by improving the capacity of mammalian cells to produce biopharmaceuticals.

## Introduction

The main components of most biopharmaceuticals are proteins produced by the recombinant DNA technology. Microbial systems are attractive for expressing certain biopharmaceutical proteins because of their low cost, high productivity, and rapid implementation^1^. However, many of these proteins are too large and complex to be produced by simple prokaryotic systems, or even in lower eukaryotic forms, such as fungi and yeasts. Complex biomolecules, like functional monoclonal antibodies or highly glycosylated proteins, need a developed post-translational machinery only available in mammalian cells^2^. Thus, they have become the dominant system for producing recombinant proteins for clinical applications. However, transforming mammalian cell lines is often time-consuming and frequently generates low-producing clones with unstable gene expression^3^. Consequently, it is required high productivity and stability of the transgene expression for developing commercially viable processes.

Transformation of mammalian cells with the desired construct predominantly uses approaches in which the gene of interest is randomly integrated into the host chromosome. The recombinant protein obtained by the transgene expression is often secreted to the culture medium, facilitating the downstream process. The specific productivity (Qp) of mammalian cell lines usually correlates with the transgene mRNA levels, which depend on the transcriptional unit design, the transgene copy number integrated into the genome, and the chromatin arrangement at the integration site^4^.

Currently, the generation of cell lines for recombinant protein production is done by two main strategies: the selection using dihydrofolate reductase (DHFR) and methotrexate (MTX)^5^ or using glutamine synthetase (GS) and methionine sulfoximine (MSX)^6^. For both systems, the selection of high-producing clones usually harbors transgenes organized in tandem arrays from tens to hundreds of copies. Hence, it is not known what is the real contribution that makes every copy of the transgene to the Qp of the selected clone. In addition, gene expression from these tandems is often unstable due to the loss of transgene copies resulting from recombination events^7–10^ or repeat-induced gene silencing^11–14^.

In this work, we used the sequential insertion of transgenes mediated by lentiviral vectors as a new method for generating stable clones producing high levels of a specific recombinant protein. We designed a bicistronic transcriptional unit for the expression of hscFSH and GFP genes, allowing the fluorescence-assisted selection of high-producing clones with a single copy of the transgen. Three whole rounds of transgene insertion-deletion were performed, showing a stepwise increasing of the hscFSH copy number together with the expression levels.

## Results

### Conceptual design

During the first stage of this work, we designed a strategy for the fluorescence-assisted sequential insertion of transgenes (FASIT). It comprised a bicistronic transcriptional unit based on the gene of interest hscFSH and an IRES sequence, followed by a gene coding the GFP protein. The last two sequences were flanked by LoxP sites with the same orientation. Consequently, a single messenger RNA (mRNA) contained the necessary information for the expression of hscFSH and GFP proteins. The translation of hscFSH and GFP genes should occur by cap-dependent and IRES-dependent mechanisms, respectively. Considering that both genes are translated from the same mRNA, it is reasonable to assume that cells showing higher fluorescence intensity will also express greater amount of the gene of interest. This approach has already been described in earlier studies, demonstrating its efficacy as a tool for selecting high-producing clones^15,16^. However, the strategy described previously has two major drawbacks: (i) the GFP accumulation inside the cells affects their viability and doubling time, due to the toxicity of GFP^17,18^, (ii) the co-expression of GFP in cell cultures could be questionable from a regulatory point of view if it is desired to start a production process with the selected clones.

**Figure 1:**
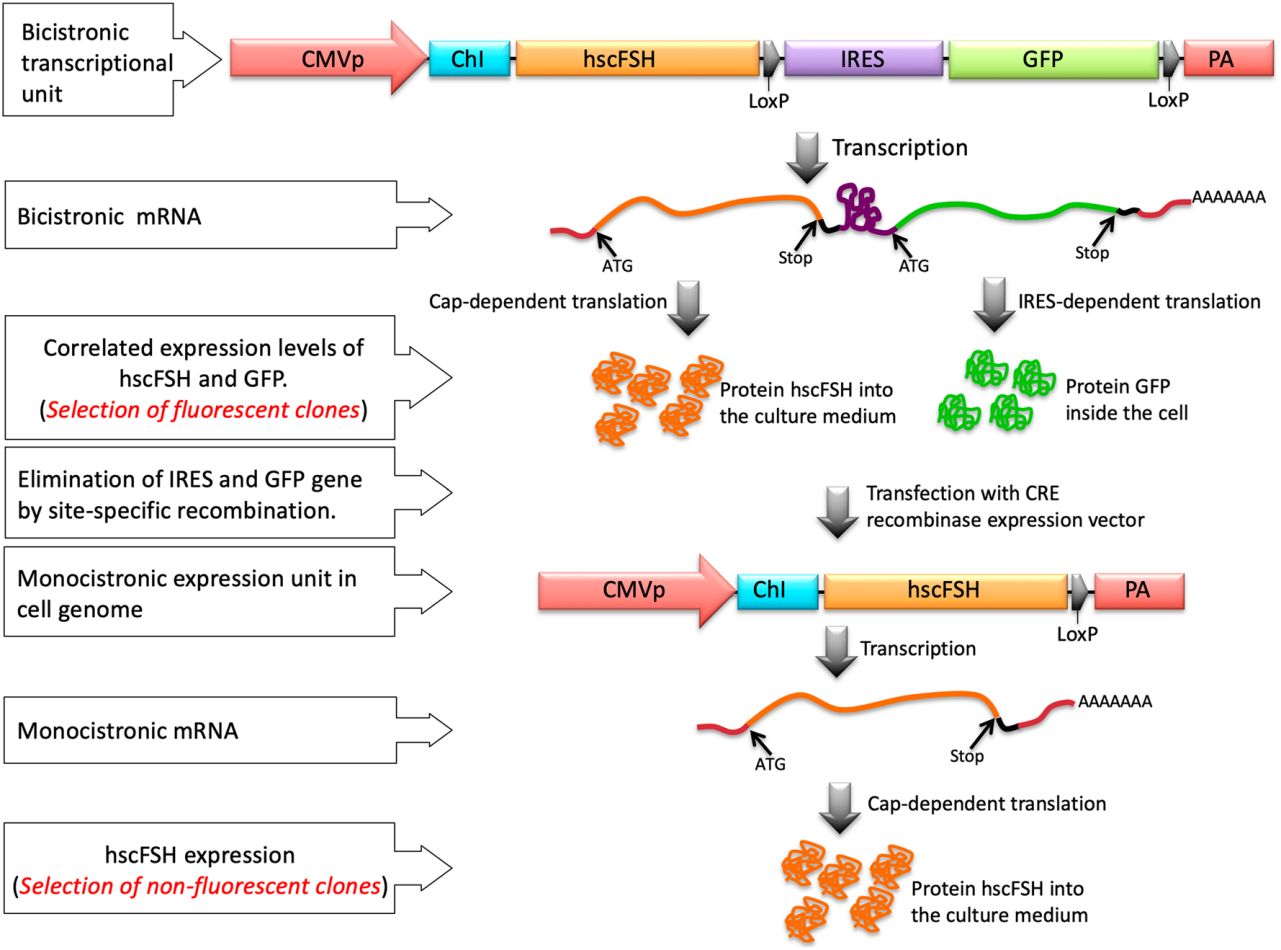
Diagram of genetic arrangements and molecular events governing the general strategy for sequential insertion of transgenes. *CMVp* early/immediate citomegalovirus promoter, *ChI* Chimeric intron, *hscFSH* human single-chain Follicle-stimulating hormone, *IRES* internal ribosome entry site, *GFP* green fluorescent protein, *PA* poly-adenylation sequence, *LoxP* Target site for the CRE recombinase.

The LoxP sites flanking the IRES and the GFP coding sequences included in our design could overcome these drawbacks. The removal of the GFP gene can be done by CRE-mediated site-specific recombination, without affecting the expression levels of the gene of interest. This recombination event should generate non-fluorescent and high-producing clones, which could be used for the insertion of additional transgene copies with the aim of increasing their Qp (Fig. 1).

### Relationship between hscFSH expression levels and fluorescence intensity

**Figure 2:**
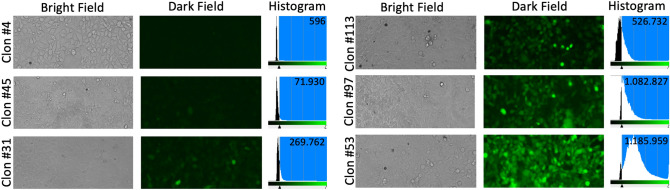
Photomicrographs and histograms of clones selected after the transfection of HEK-293 cells with the plasmid pEntry-hscFSH. Variable expression levels of GFP were detected in the different clones by observation at the fluorescence microscope.

We demonstrated the direct relationship between hscFSH expression levels and fluorescence intensity by transfecting HEK-293 cells with the plasmid pEntry-hscFSH. Stably transformed clones were selected with G418 in 100 mm plates. A total of 122 stably transformed clones were obtained from six plates, which were analyzed by diameter and fluorescence level (Supplementary Table 1). Six clones showing variable levels of fluorescence were selected and expanded. Figure 2 shows bright and dark field photomicrographs for every amplified clone. Histograms display the number and intensity of green pixels resulting from the GFP expression. A clear shift to the right of the histograms was observed, which coincides with the intensity observed in dark field photomicrographs.

**Figure 3:**
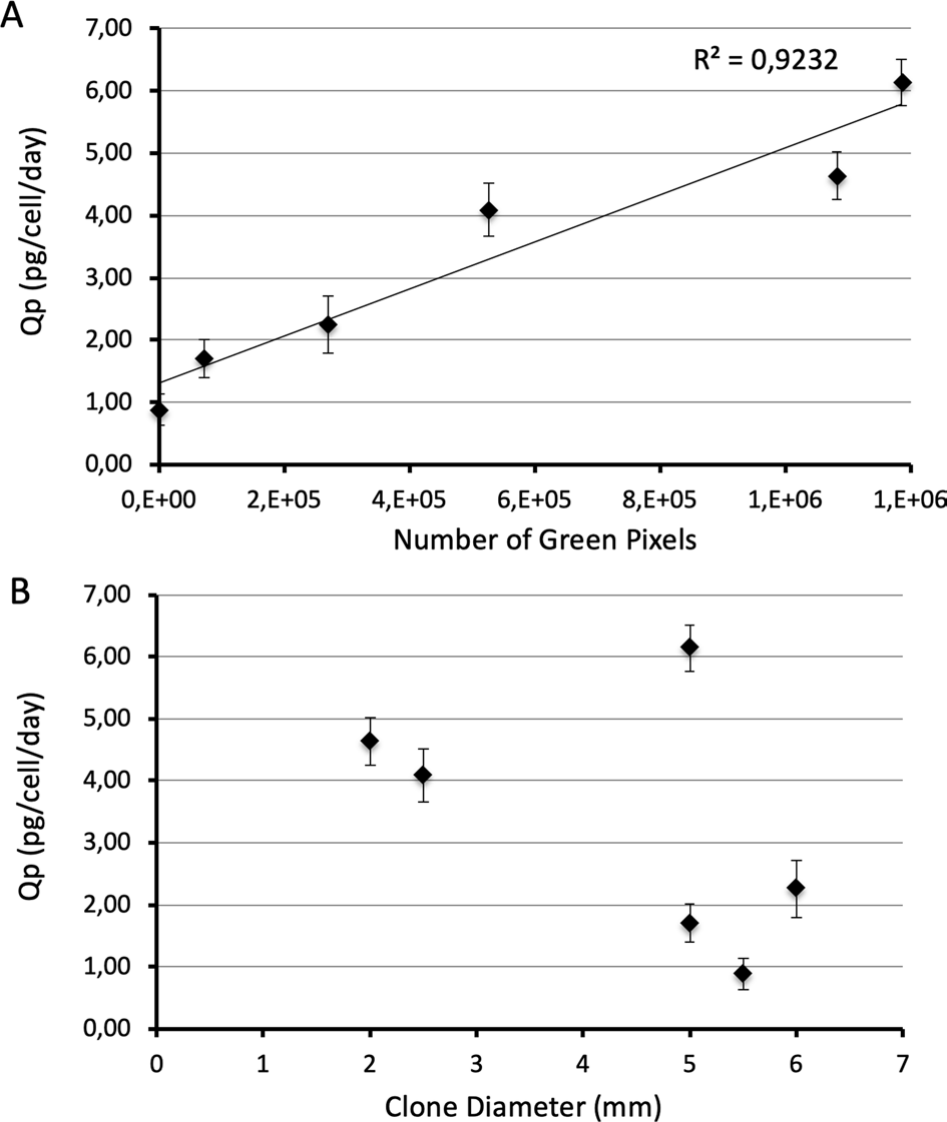
Relationship among the Qp of hscFSH, the GFP expression levels, and the size of clones after their selection with G418. (**A**) Association between the Qp of hscFSH and the fluorescence intensity designated as number of green pixels. (**B**) Relationship between the Qp of hscFSH and the clone diameter. Bars represent the standard deviation.

The hscFSH Qp, fluorescence intensity and clone diameter were determined for the six clones selected. The Qp ranged between 0.88 and 6.14 pg/cell/day, showing a relationship between the fluorescence intensity and the hscFSH concentration (Fig. 3A). However, no association was observed between the Qp and the clone diameter (Fig. 3B), which indicates that best proliferating clones under the selective pressure of G418 are not necessarily those where the transgene is best expressed.

### Insertion of the first transgene

**Figure 4:**
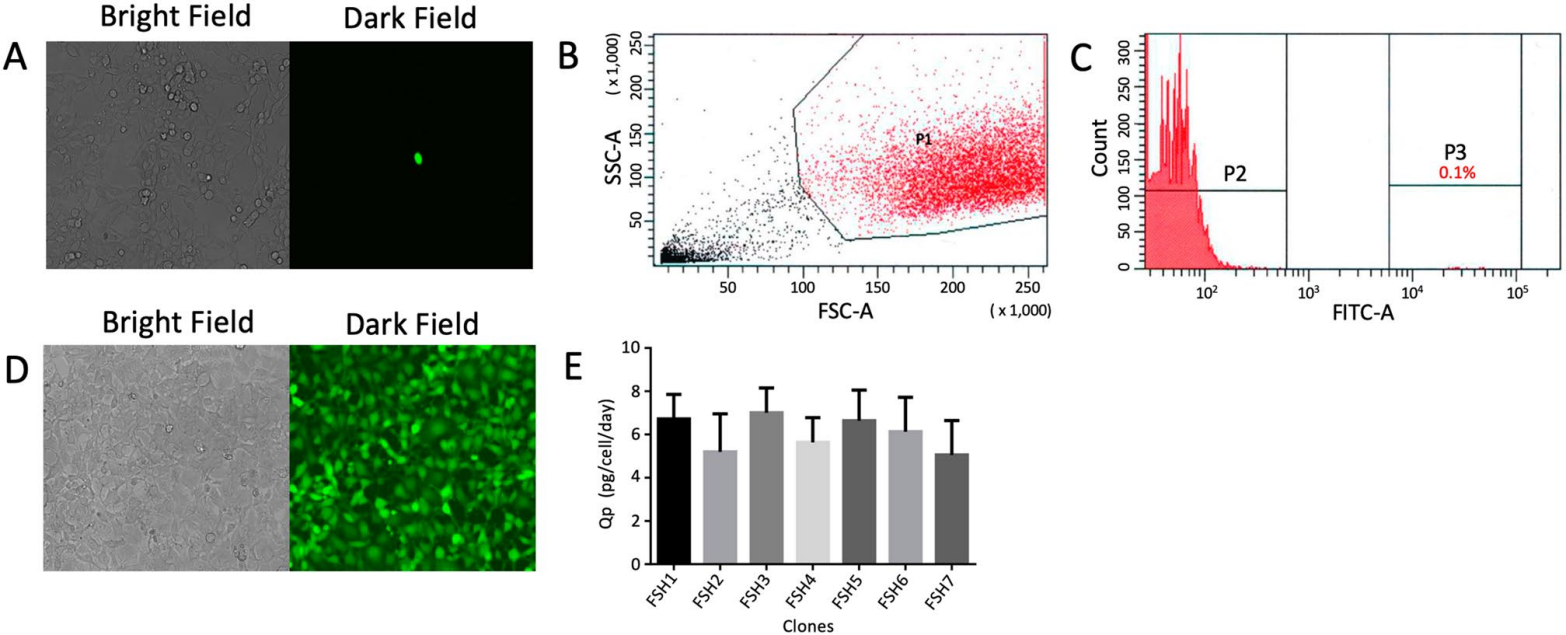
Insertion of the first hscFSH copy by lentiviral transduction. (**A**) Bright field and dark field photomicrographs of HEK-293 cells transduced with the lentiviral vector LCW-hscFSH at a MOI of 0.01. (**B**) Forward versus side scatter plots of HEK-293 cells transduced with the lentiviral vector LCW-hscFSH. Cells were gated (P1) and analyzed for GFP expression. (**C**) Histogram of HEK-293 cells expressing GFP. Highly fluorescent cells (P1) were sorted directly into a 96 well plate. (**D**) Bright and dark field photomicrographs of the clone FSH3 selected by flow cytometry and cell sorting. (**E**) Qp of hscFSH from seven fluorescent clones. Bars represent the standard deviation. Qp values from different clones were compared by the Kruskal–Wallis test and the Dunn post-test.

Stable insertion of the first transgene was done by transducing HEK-293 cells with the lentiviral vector LCW-hscFSH in a single well of a 96-well plate. In this assay, a MOI of 0.01 (one infective viral particle per 100 cells) was used to ensure that every cell was transduced by a single viral particle. Figure 4A shows a single fluorescent cell in the dark field after 48 h of transduction. Next, cells were grown at 70–80% of confluence and submitted to flow cytometry and cell sorting. The SSC vs FSC density plot, with a gate applied to the cell population of interest, allowed the quantification of the number of cells with detectable levels of fluorescence in 0.6% (Fig. 4B). Figure 4C shows the histograms of GFP expression and the sorting gate (P3) containing the brightest fluorescent cells. Individual sorted cells were transferred to 96-well plates.

After a week of culture, seven wells containing single fluorescent clones were selected and named from FSH1 to FSH7. These clones were amplified and observed at the fluorescence microscope. A hundred percent of cells from the clone FSH3 were fluorescent (Fig. 4D). Interestingly, the seven clones showed a Qp between 5 and 7 pg/cell/day with no significant differences between them (Fig. 4E).

### Deletion of the fluorescent marker

**Figure 5:**
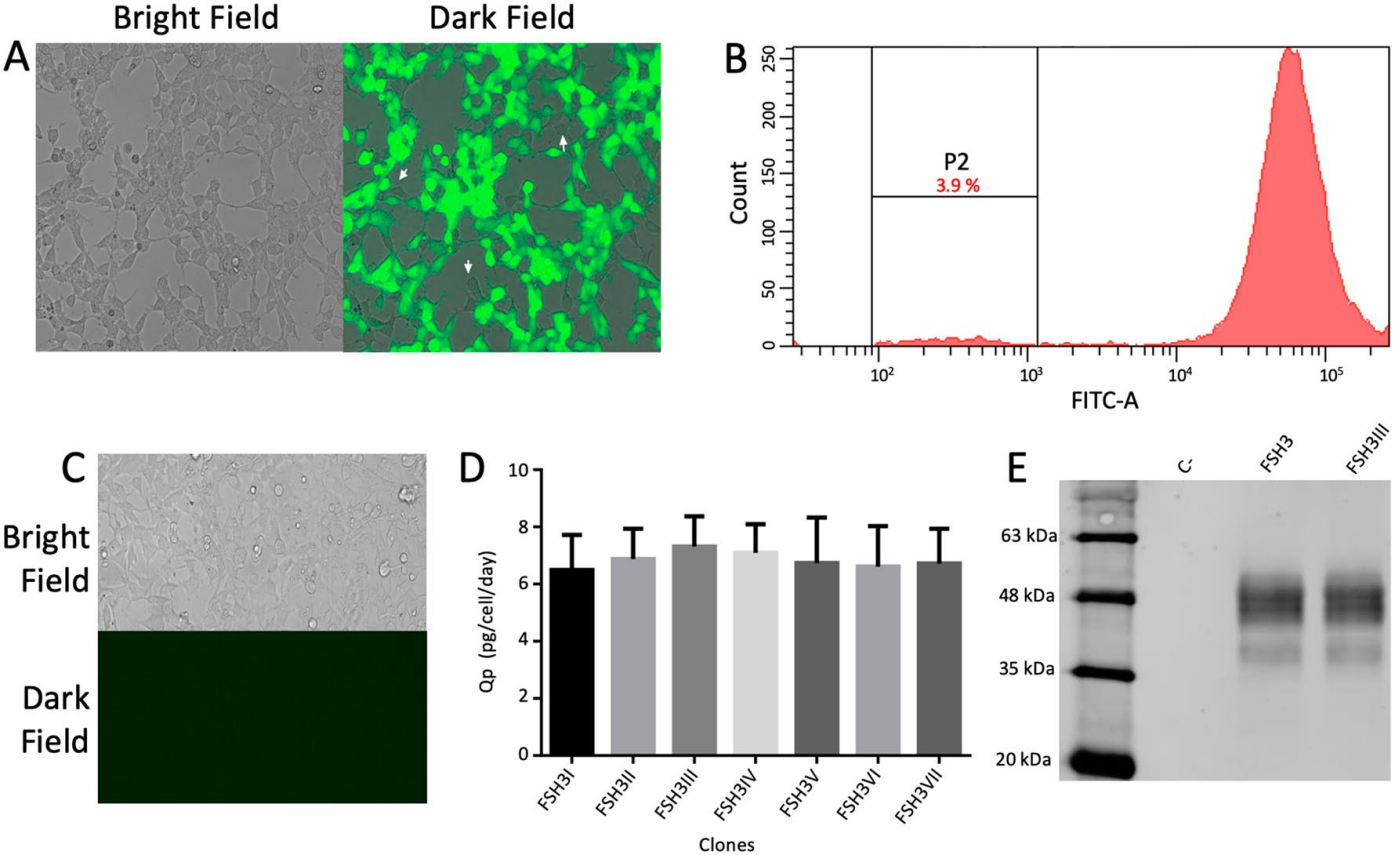
Removal of GFP gene by site-specific recombination using the recombinase CRE. (**A**) Bright and dark field photomicrographs of the clone FSH3 transfected with the plasmid pAEC-CRE. Arrows in the dark field show cells without GFP expression. (**B**) Analysis of the GFP expression by flow cytometry. The sorted gate is indicated as P2. (**C**) Bright and dark field photomicrographs of the non-fluorescent clone FSH3III selected by flow cytometry and cell sorting. (**D**) Qp of hscFSH from seven non-fluorescent clones. Bars represent the standard deviation. Qp values from different clones were compared by the Kruskal–Wallis test and the Dunn post-test. (**E**) Western-blot of the hscFSH protein from the clone FSH3 before and after removing the GFP gene (clone FSH3III). Non-transduced cells were used as a negative control (C−). The reaction was visualized using the imaging system ODYSSEY CLx and the software Image Studio version 3.1 (https://www.licor.com/bio/image-studio-lite/download).

The clone FSH3 was selected from the previous experiment to continue the next round of modification. It was amplified and frozen (five vials), for further expression analysis. To remove the GFP sequence, the selected clone was transfected with the plasmid pAEC-CRE. After 96 h, the presence of non-fluorescent cells was visualized, suggesting the depletion of the GFP gene (Fig. 5A). A flow cytometry coupled to cell sorting assay allowed the selection of non-fluorescent cells, which constituted the 3.9% of the total cells (Fig. 5B). These cells were separated in a 96-well plate and 7 subclones, all derived from the clone FSH3, were expanded and designated from FSH3I to FSH3VII. The dark field photomicrograph corresponding to clone FSH3III showed that 100% of cells were non-fluorescent (Fig. 5C).

The Qp determination of all subclones showed levels of hscFSH around 7 pg/cell/day (Fig. 5D). This value of Qp was similar to the previously obtained from the original fluorescent clone FSH3. Also, a Western-blot assay showed immunoreactive bands around 48 kDa with similar intensity, corresponding to the hscFSH expression by the original clone FSH3 and the non-fluorescent subclone FSH3III. Additional bands of about 40 kDa were detected, which could correspond to a degradation product or to a partially glycosylated form of hscFSH (Fig. 5E). These issues confirm the concept that the GFP coding sequence can be removed without affecting the expression levels of the gene of interest.

### Incorporation of additional copies of the transgene

**Figure 6:**
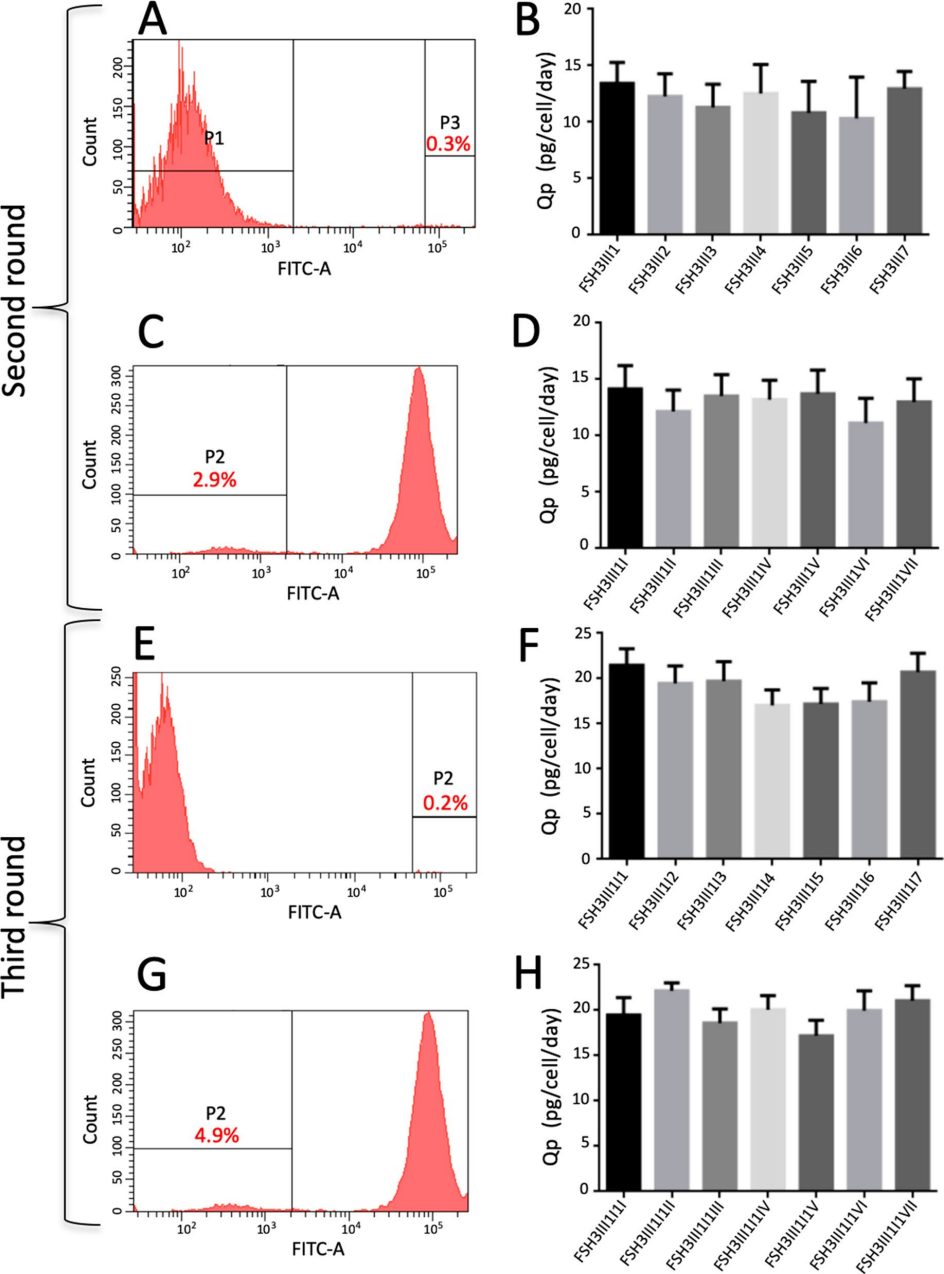
Sequential insertion and deletion of transgenes from the HEK-293 genome. The FSH3III clone was subjected to two additional rounds of transgene insertion. Histograms of the GFP expression are shown in panels **A**, **C**, **E** and **G**. The gate and the percentage of sorted cells are shown. Seven clones were amplified in every round of selection. The Qp of these clones is shown in graphs **B**, **D**, **F** and **H**. Bars represent the standard deviation. Qp values from different clones were compared by the Kruskal–Wallis test and the Dunn post-test.

The clone FSH3III was chosen for adding one more copy of the hscFSH gene. The cells underwent a second round of transduction with the lentiviral vector LCW-hscFSH, which yielded 0.3% of fluorescent cells (Fig. 6A). Seven clones with the highest levels of fluorescence were expanded and their Qp was determined. It was observed that all clones expressed hscFSH between 11 and 14 pg/cell/day (Fig. 6B). The nomenclature of the clones established above was continued. Thus, every subclone has the name of the preceding clone. The name ends in Arabic number if the clone is fluorescent, or in Roman number, if the GFP gene has been removed.

From the second round of transduction, the clone FSH3III1 was selected for removing the fluorescent marker by site-specific recombination. The transfection of cells with the plasmid pAEC-CRE yielded 2.9% of non-fluorescent cells (Fig. 6C). Seven of the non-fluorescent clones were expanded and analyzed for hscFSH expression. The clones showed Qp values of about 14 pg/cell/day, which were very similar to that of the parental clone FSH3III1 (Fig. 6D).

The third round of transduction was done with the clone FSH3III1I. After a new round of transduction with the lentiviral vector LCW-hscFSH, 0.2% of fluorescent cells were obtained (Fig. 6E). Again, seven clones with the highest levels of fluorescence were isolated and amplified. The Qp of these clones was higher than that of the clones with two rounds of transduction. The expression levels ranged between 17 and 21.5 pg/cell/day, which is consistent with the incorporation of a new copy of the hscFSH gene (Fig. 6F). The clone FSH3III1I1 was selected for removing the fluorescence marker. The transfection of this clone with the plasmid pAEC-CRE yielded 4.9% of non-fluorescent cells (Fig. 6G). As in the previous rounds, seven non-fluorescent clones were isolated and amplified. The Qp of these clones was around 20 pg/cell/day (Fig. 6H). At this stage, the clone FSH3III1I1II was selected for studying the relationship between the number of transduction rounds and the expression levels of hscFSH.

### Relationship between the transduction rounds and the expression levels

**Figure 7:**
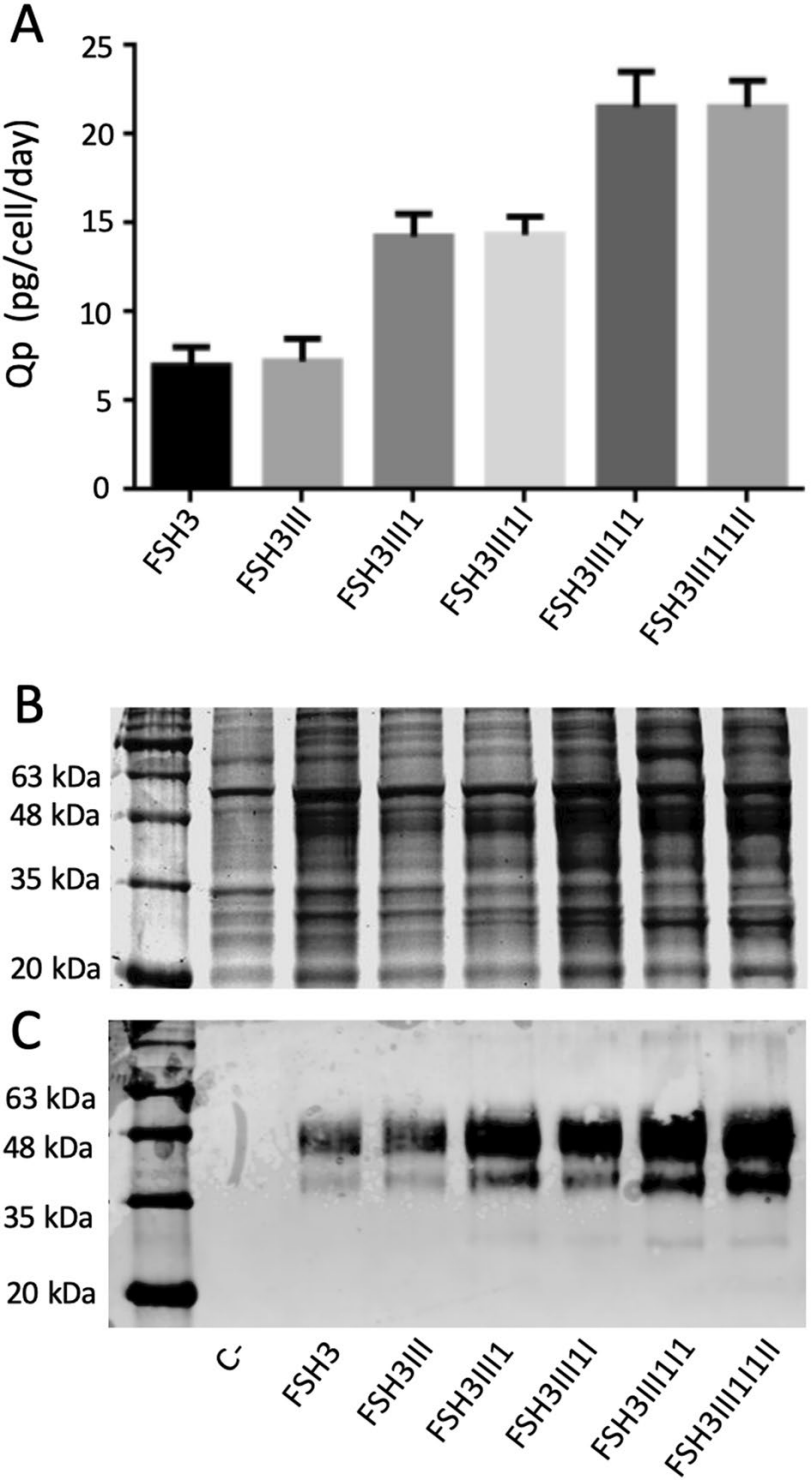
Increase of the hscFSH expression levels in clones with several rounds of lentiviral transduction. (**A**) Quantification of hscFSH Qp from three clones with one, two or three rounds of hscFSH gene insertion and GFP gene deletion. Bars represent the standard deviation. Qp values corresponding to the clones with and without the fluorescent marker were compared by the Mann Whitney test. Detection of the hscFSH protein by SDS-PAGE (**B**) and Western-blot (**C**) from the supernatant of the previous clones. The hscFSH protein was immunoidentified using a mouse anti-human FSH-beta antibody and a goat anti-mouse IgG [H&L] conjugated to IRDye 800. Non-transduced cells were used as a negative control (C−). The reaction was visualized using the imaging system ODYSSEY CLx and the software Image Studio version 3.1 (https://www.licor.com/bio/image-studio-lite/download).

The clones selected in the different rounds of genetic manipulation were thawed and amplified to study the Qp variations. The Qp was determined for the clones FSH3, FSH3III, FSH3III1, FSH3III1I, FSH3III1I1, and FSH3III1I1II. After every round of transduction with LCW-hscFSH, the levels of hscFSH expression raised around 7 pg/cell/day. The transfection with the vector pAEC-CRE for the removal of the fluorescence marker, does not affect the hscFSH expression levels of the preceding clone (Fig. 7A).

The presence of the hscFSH protein in the culture medium of each selected clone was evaluated by SDS-PAGE and Western-blot. Immunoreactive bands of around 48 kDa were observed, which is consistent with the previous assay. The band corresponding to the hscFSH protein in the clones derived from the second and third rounds of transduction can even be distinguished in the SDS-PAGE (Fig. 7B,C). The clone FSH3III1I1II was selected for the production of the chimeric protein hscFSH. This clone has been used in our laboratory for more than two years with Qp evaluations every six months. So far, these evaluations have shown Qp values around 21 pg/cell/day.

## Discussion

The production of biopharmaceuticals in mammalian cell cultures initially involves the insertion of the transgene of interest into the host cells, which subsequently secrete the synthesized product to the culture medium, and it is obtained in its pure form by performing an optimal downstream process. The volumetric productivity of these mammalian cell cultures depends on two fundamental variables: (i) the Qp expressed as the amount of protein produced by a single cell in a day [pg/cell/day], and (ii) the concentration of these cells in a given volume [cell/mL].

During the last 20 years, significant progress has been made in the development of culture media and bioreactor systems with the aim of increasing the cell concentration during the upstream processes^19^. However, little has been done for developing strategies to increase the Qp. Current cell lines are generated using the selection systems based on DHFR/MTX or GS/MSX. The high-producing clones selected by both systems usually insert the transgene in head-to-tail tandem arrays. Gene expression from these kinds of gene arrangements is often unstable due to the loss of transgene copies by recombination events or by repeat-induced gene silencing.

We decided to explore a novel strategy for generating cell lines with high Qp of the product of interest. We took advantage of the lentiviral vector system, which inserts foreign genes into transcriptionally active regions of the host genome mediated by viral integrases^20^. This mechanism does not allow the formation of tandem repeats. Thus, it is unlikely to lose copies of the transgene due to recombination events.

In this study, we demonstrated the correlation between the expression levels of GFP and hscFSH genes after their translation from a single bicistronic RNA. This relationship suggests the possibility of selecting high-producing clones based on their fluorescence level. The selection could be made by flow cytometry coupled to cell sorting, as described in this work, or by micro-trypsinization of the most fluorescent clones selected after a simple observation under a fluorescence microscope. In fact, the micro-trypsinization technique, using Scienceware Cloning Discs (Merck, catalog no. Z374431), has been widely used in our laboratory during the last months, allowing the generation of multiple stable lines without the need of expensive equipment, such as the flow cytometer.

The possibility of eliminating the fluorescence marker by site-specific recombination is, in our opinion, one of the most outstanding features of this work. The GFP gene is only used for the selection of high-producing clones. Once they are isolated and characterized, it is possible to eliminate the reporter gene without affecting the expression levels of the gene of interest. This strategy has two fundamental advantages (i): high-producing clones will not express any additional protein but the gene of interest, which make this approach suitable for producing biopharmaceuticals with a more expedited transit through regulatory barriers, and (ii): the selected clone can be reused to incorporate new copies of the transgene for increasing its Qp in a step-wise manner.

A curious finding of investigation was the increment of the Qp of high-producing clones by approximately 7 pg/cell/day after every round of transduction. It seems that it is the highest expression level provided by the cell transduction with a single infective particle of the lentiviral vector LCW-hscFSH. Unfortunately, we cannot state for sure that the increase of about 7 pg/cell/day is the result of a single event of integration. It should be considered that every lentiviral particle carries two viral genomes. To date, it is not known whether both genomes or only one is retrotranscribed and inserted into the host cell genome. Our attempts to quantify the number of transgene copies in the selected clones, by qPCR and Southern-blot, were inconclusive. But, whatever the number of transgene copies (one or two) is integrated after the entry of a single lentiviral particle into the cell, the rising on the expression levels was always discrete and relatively constant in every round of transduction. This fact has been corroborated several times in our laboratory when the same technique has been used for generating cell lines expressing recombinant single chain antibodies.

The cell lines mentioned above have been used in our laboratory for more than two years with no detectable loss of the expression levels, suggesting that the insertion of individual copies of the transgene into different regions of the genome could significantly reduce silencing due to recombination; and it could significantly reduce, if not avoid entirely the repeat-induced gene silencing. Despite these advantages, we cannot claim that this strategy allows the elimination of any form of epigenetic silencing. It is widely documented that epigenetic silencing is not only caused by tandem repeats. It also is induced by the very frequent methylation of the CpG dinucleotides inside the promoters^21,22^.

The methodology described here is robust, reproducible, and could be implemented in any cell type permissive to lentiviral vectors. The extent of this technique is still unknown; since additional copies of the transgene could be incorporated until saturation of the cell transcriptional and translational machinery, with the probable loss of cell viability. Currently, our work is aimed to find the maximum copy number or expression levels that a CHO cell line supports for producing a chimeric variant of bovine FSH.

The main limitation of this technique lies in the time required for each round of transduction and the elimination of the marker gene. Each genetic manipulation takes about a month, meaning that it is required approximately 60 days for performing the lentiviral transduction and removing the marker gene. Thus, about 6 rounds of transduction could be done in a year of continuous genetic manipulation. If each round contributed to an increase of 7 pg/cell/day, the Qp of the clones could be around 50 pg/cell/day. But still, one question needs to be answered. Is it possible to increase the Qp of clones to the desired value of 100 pg/cell/day by performing additional rounds of transduction?

Overall, we designed and evaluated a new strategy to generate high-producing cell lines. Supplementary figure S2 describes the FASIT methodology. The procedure allows the sequential insertion of transgene copies, with the consequent increase of the expression levels of the desired gene. High-producing clones can be easily identified and selected by flow cytometry coupled to cell sorting or even by micro-trypsinization. This methodology allows to know the contribution that every transgene integration event makes to the gene expression, which should maximize the ratio Qp/number of integrated copies. The procedure could not only impact in the biopharmaceutical industry as a tool for generating high-producing clones, but also constitutes a powerful instrument for studying the Qp limits that have the conventional cell lines used for producing biopharmaceuticals at industrial scale.

## Materials and methods

### Plasmid construction

A single-chain human follicle-stimulating hormone variant (hscFSH) was designed as the protein of interest. Alpha and beta subunits of this molecule were covalently linked by a 16 amino-acid spacer peptide. A tag of six histidine residues was added at the C-terminus. Based on the amino acid sequence, the hscFSH would have a theoretical molecular weight of 28.8 kDa. However, the hscFSH has 5 potential N-glycosylation sites. Four of them are already included in the native sequence, while the fifth was incorporated into the spacer peptide. Thus, if the 5 potential N-glycosylation sites are occupied and it is estimated a weight of 4 kDa for each N-linked oligosaccharide chain^23,24^, the final size of the hscFSH should be around 48 kDa.

We designed a bicistronic DNA sequence containing the following elements: the hscFSH coding sequence, a 5′-3′ LoxP site, an Internal Ribosome Entry sequence (IRES), the Green Fluorescent Protein (GFP) gene, and a second 5′-3′ LoxP site. After gene synthesis (Blue Heron Biotech, USA), the sequence was inserted into the XhoI/EcoRV site of the mammalian expression vector pEntry-CMV (Origene Technologies, USA), obtaining the plasmid pEntry-hscFSH (Supplementary Fig. S1A).

The plasmid pLW^25^ was used as transfer vector for the obtaining of a lentiviral vector expressing the hscFSH gene. This plasmid was digested with the restriction enzyme NheI (New England Biolabs, USA) and protruding ends were blunted with the Mung Bean nuclease (New England Biolabs, USA) for further dephosphorylation with the Shrimp Alkaline Phosphatase (New England Biolabs, USA). A DNA segment containing the CMV promoter and a chimeric intron was removed from the pTarget vector (PROMEGA, USA), by digesting with MscI and NheI (New England Biolabs, USA). The digested product was treated with the Klenow fragment (New England Biolabs, USA) to fill the overhang ends generated by NheI. The blunt DNA segment was then cloned into the plasmid pLW to generate the plasmid pLCW. The insertion of the bicistronic DNA sequence into the pLCW vector was done by digesting the plasmids pLCW and pEntry-hscFSH, with XhoI (New England Biolabs, USA) and EcoRV (New England Biolabs, USA). The purified DNA fragments were ligated to obtain the final construction pLCW-hscFSH (Supplementary Fig. S1B).

### Generation and titration of the lentiviral vector

The lentiviral vector LCW-hscFSH was generated by co-transfecting 293 T cells (ATCC CRL-3216) with the plasmid pLCW-hscFSH, together with three packaging plasmids: pLP1, pLP2, and pLP/VSVG (Thermo Fisher Scientific, USA). Transfection was performed using the polyethylenimine-based method described by Toledo and coworkers in 2009^25^. Specifically, 293 T cells were transfected for 8 h in 100 mm plates (Corning, USA) with 20 μg of DNA using the following plasmid rate: 8 μg of pLCW-hscFSH, 4 μg of pLP1, 4 μg of pLP2, and 4 μg of pLP/VSVG. The supernatant was harvested 48 h post-transfection. After filtering the supernatant through 0.45 μm pore size, virus was concentrated by ultracentrifugation at 80 000 g for 1.5 h. The resulting pellet was resuspended in 80 μL of PBS (0.137 M NaCl, 0.003 M KCl, 0.008 M Na_2_HPO_4_, and 0.001 M NaH_2_PO_4_ (pH 7.4)). Lentiviral preparation was aliquoted and stored at -80 °C until further use. Lentiviral titration was done by counting gene transfer units (GTU) or transducing particles, using the reporter gene GFP, in HEK-293 cells (ATCC CRL-1573). 96-well plates (Corning, USA) were seeded with HEK-293 cells at 2 × 10^4^ cells/well and transduced with the lentiviral vector by making dilutions 1:5 in DMEM medium until completing the whole plates. The last column of the 96-well plates was not transduced with the lentiviral vector and therefore, it was used as negative control. Evaluation of GFP marker was done 72 h later in a fluorescence microscope. Titration values were considered for the last dilution where green cells were observed. Each titration included four replicates.

### Generation of stable cell lines by plasmid transfection

HEK-293 cells were transfected with the plasmid pEntry-hscFSH, previously digested with AgeI (New England Biolabs, USA). Transfection was performed in 100 mm plates using the polyethylenimine-based method mentioned above. Two days after transfecting with 20 µg of DNA per plate, the cells were trypsinized (Sigma, USA) and plated for G418 selection (500 μg/ml) (InvivoGen, USA). Fresh G418-containing medium was added every 4 days. Colonies resistant to G418 were visible after 20 days. Fluorescence was observed in dark fields using an excitation filter of 482/18 nm and an emission filter of 532/59 nm. The size of clones was measured, and the fluorescence intensity was quantified using the Photoshop program as described below.

### Generation of stable cell lines by lentiviral transduction

Once the titer of LCW-hscFSH was determined, we obtained stable clones by transducing the cells in a single well of a 96-well plate at a multiplicity of infection (MOI) of 0.01. The low MOI ensures that every transduced cell incorporates only one viral particle. After 48 h, the presence of fluorescent cells was visualized at the fluorescence microscope. The transduced culture was then transferred to a well of a 24-well plate (Corning, USA). After reaching confluence, it was transferred again to a 25 cm^2^ culture flask (Corning, USA). Finally, the cells were treated with trypsin and seeded into two 100 mm dishes. The cells of one of these dishes were frozen and the other cells were used for flow cytometry and cell sorting assays.

### Cell sorting

Cell sorting was performed in a BD FACSAria cell sorter (Becton Dickinson, USA) equipped with an automatic cell deposition unit for sorting into plates. Cells were treated with trypsin, washed with PBS and strained through a 70 μm nylon mesh prior to analysis. The GFP protein was excited with a 13–20 mW Coherent Sapphire solid-state laser at 488 nm, and the emission was collected with a HQ 510/20-BP filter. Routinely, 30,000 events are acquired for each sample. Debris and doublets were excluded by gating on forward and side scatter dot plots (FSC-A vs. SSC-A, FSC-W vs FSC-H and SSC-W vs. SSC-H). Cells within these gates were examined for GFP expression. The cells with the highest levels of fluorescence were sorted after every round of transduction with LCW-hscFSH. In contrast, the cells with no detectable levels of fluorescence were separated after every round of transfection with the plasmid carrying the CRE recombinase gene (see below). After cell sorting, the cells were maintained in 96-well plates for at least 2 weeks until the formation of clones. Subsequently, the individual clones were transferred to 24-well plates and analyzed for hscFSH expression.

### Excision of the GFP coding sequence

Fluorescent clones derived from LCW-hscFSH transduction were transfected with the plasmid pAEC-CRE (Supplementary Fig. S1C), containing the gene coding for the CRE recombinase under the control of a CMV promoter. Transfection was performed in 25 cm^2^ culture flasks using the polyethylenimine-based method mentioned above with 5 µg of DNA per flask. After 48 h, cells were treated with trypsin, seeded into a 100-mm dish and cultured for another 48 h. The presence of non-fluorescent cells indicated a successful site-specific recombination event, with the consequent excision of the IRES-GFP sequence.

### Determination of fluorescence intensity

The fluorescence intensity in the stably transformed clones was determined by direct observation in an EVOS FLoid Cell Imaging Station (Thermo Fisher Scientific, USA). We made an arbitrary quantification of the fluorescence in every preselected clone by counting the number of green pixels. At least three photomicrographs of each clone were taken and analyzed in the Photoshop program using the option “Histogram”. In the window “Channel” we select “green”, which shows all the green tones (from 0 [near to black] to 255 [bright green, near to white]). We marked the point 255 inside the histogram with the left click of the mouse, and crawl the mouse up to the desire cut value (we established 50). This action allows counting all the pixels inside the picture with a green tonality between 50 and 255. The resulting histogram was similar to that obtained by a flow cytometer, and the number of green pixels resembled the number of positive events observed in a cytometry assay.

### Detection and quantification of hscFSH protein

For SDS-PAGE and Western blot assays, HEK-293 cells containing the hscFSH gene were cultured in 100 mm plates with DMEM medium (Sigma, USA) and 10% fetal bovine serum (Biological Industries, Israel). After reaching confluence, cells were washed twice in PBS and incubate with serum-free medium for 72 h. One mL of the supernatant was precipitated with trichloroacetic acid and analyzed by SDS-PAGE and Western blot. SDS-PAGE assay was done as described by Laemmli in 1970^26^, using 12% polyacrylamide gels. For Western blot, proteins were transferred to a nitrocellulose blotting membrane (GE Healthcare, Sweden) using a semidry electroblotter (Bio-Rad, USA). The protein hscFSH was detected using a mouse anti-human FSH-beta antibody (OriGene, catalog no. TA501624) as a primary antibody, followed by incubation with a goat anti-mouse IgG [H&L] conjugated to IRDye 800CW (Abcam, catalog no. Ab216772). The reaction was visualized using the imaging system ODYSSEY CLx and the software Image Studio version 3.1 (LI-COR Biosciences, USA).

For measuring the specific productivity (Qp), the selected clones were seeded in 12-well plates (Corning, USA) at a density of 1 × 10^5^ cells/well. A total of 4 wells per clone were seeded for further analysis. After reaching confluence (approximately 48 h later), one well from each clone was treated with trypsin and the viable cells (C1) were counted. The remaining three wells were washed twice with PBS and incubated in serum-free medium for 24 h. The supernatant was collected and the cells were treated with trypsin for counting the number of viable cells in each well (C2). The concentration of hscFSH in the supernatant was determined by the DIAsource FSH ELISA Kit (DIAsource ImmunoAssays, catalog no. KAPD1288). The Qp was calculated using the following formula: Qp = P / ([C1 + C2] / 2), where P is the amount of FSH contained in 1 mL of culture medium.

### Statistics

The statistical calculation was done with the statistical software GraphPad Prism version 8.3.1 (GraphPad Software, Inc., USA). Qp values from different clones were compared by the Kruskal–Wallis test and the Dunn post-test. Qp values corresponding to the clones with and without the fluorescent marker (Fig. 7A) were compared by the Mann Whitney test. Significance was considered for *p* < 0.05.

## Supplementary information

Supplementary file1 (DOCX 32783 kb)
