# Supplementary material for: Fluorescence-assisted sequential insertion of transgenes (FASIT): an approach for increasing specific productivity in mammalian cells
Source: Sci Rep. 2020 Jul 30;10:12840. doi: 10.1038/s41598-020-69709-1 (PMC7392891; doi:10.1038/s41598-020-69709-1)
Supplement: Supplementary file 1 — Supplementary file1 (DOCX 32783 kb) [file 41598_2020_69709_MOESM1_ESM.docx]

**Supplementary information**

**Title:** Fluorescence-assisted sequential insertion of transgenes: an approach for increasing specific productivity in mammalian cells.

**Authors:** Felipe E. Bravo, Natalie Parra, Frank Camacho, Jannel Acosta, Alaín González, Jorge R. Toledo, Oliberto Sánchez

**Supplementary Figure S1**

**
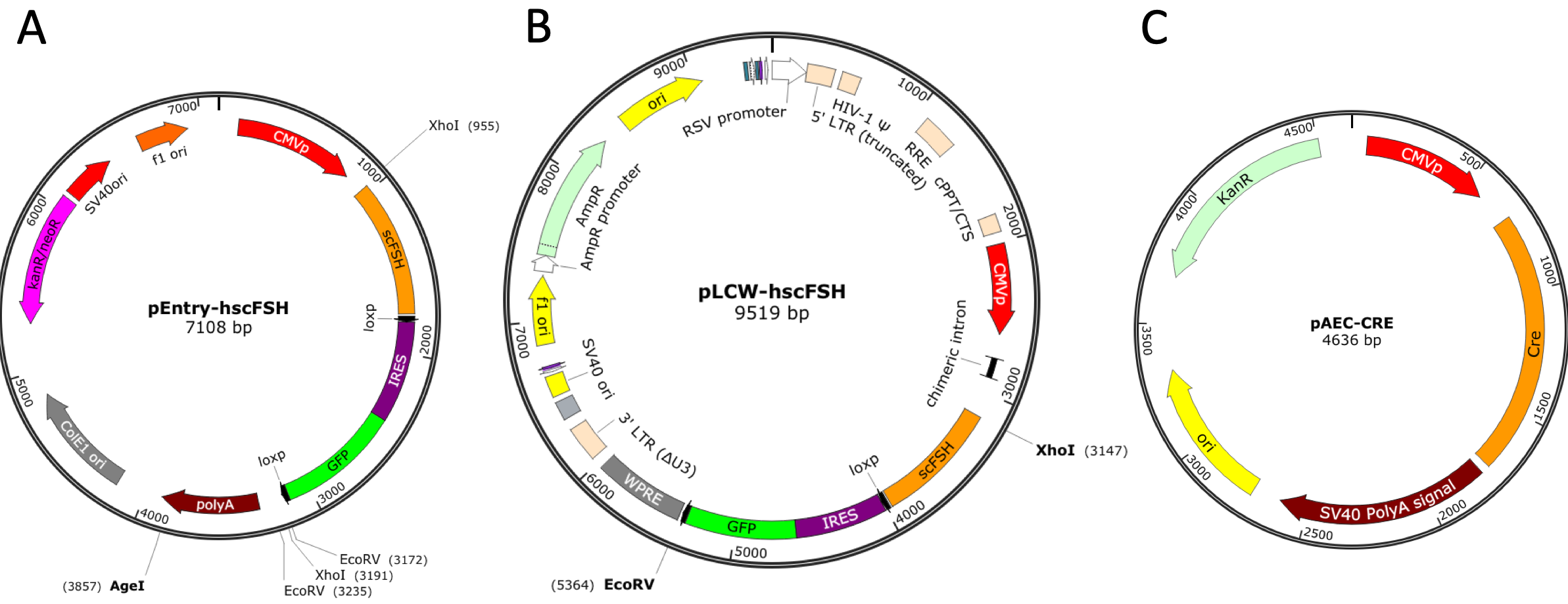
**

**Supplementary fig. S1:** Main plasmids used in this work: (A) Plasmid pEntry-hscFSH. It has a bicistronic DNA sequence with the following elements: the coding sequence for the hscFSH gene, a LoxP site, an Internal Ribosome Entry sequence (IRES), the Green Fluorescent Protein (EGFP) gene, and a second LoxP site. Both LoxP sites have the same orientation. The sequence is under the control of the cytomegalovirus (CMV) promoter and is followed by the bGH poly(A) signal. The pEntry-hscFSH also has a kanamycin/neomycin phosphotransferase expression cassette for providing resistance to G418. (B) Plasmid pLCW: This is a third-generation lentiviral transfer plasmid. It has the bicistronic DNA sequence under the control of the CMV promoter, followed by a woodchuck hepatitis virus posttranscriptional regulatory element (WPRE). The pLCW also has the HIV-1 packaging signal and other sequences necessary for reverse transcription and vector integration. (C) Plasmid pAEC-CRE: The plasmid has the coding sequence of the CRE recombinase gene under the control of a CMV promoter. The gene is followed by an SV40 bidirectional poly(A) signal

**Supplementary table 1:** Size and fluorescence level of clones stably transformed with the vector p Entry-hscFSH. Clones amplified and analyzed for hscFSH expression are shown in gray.


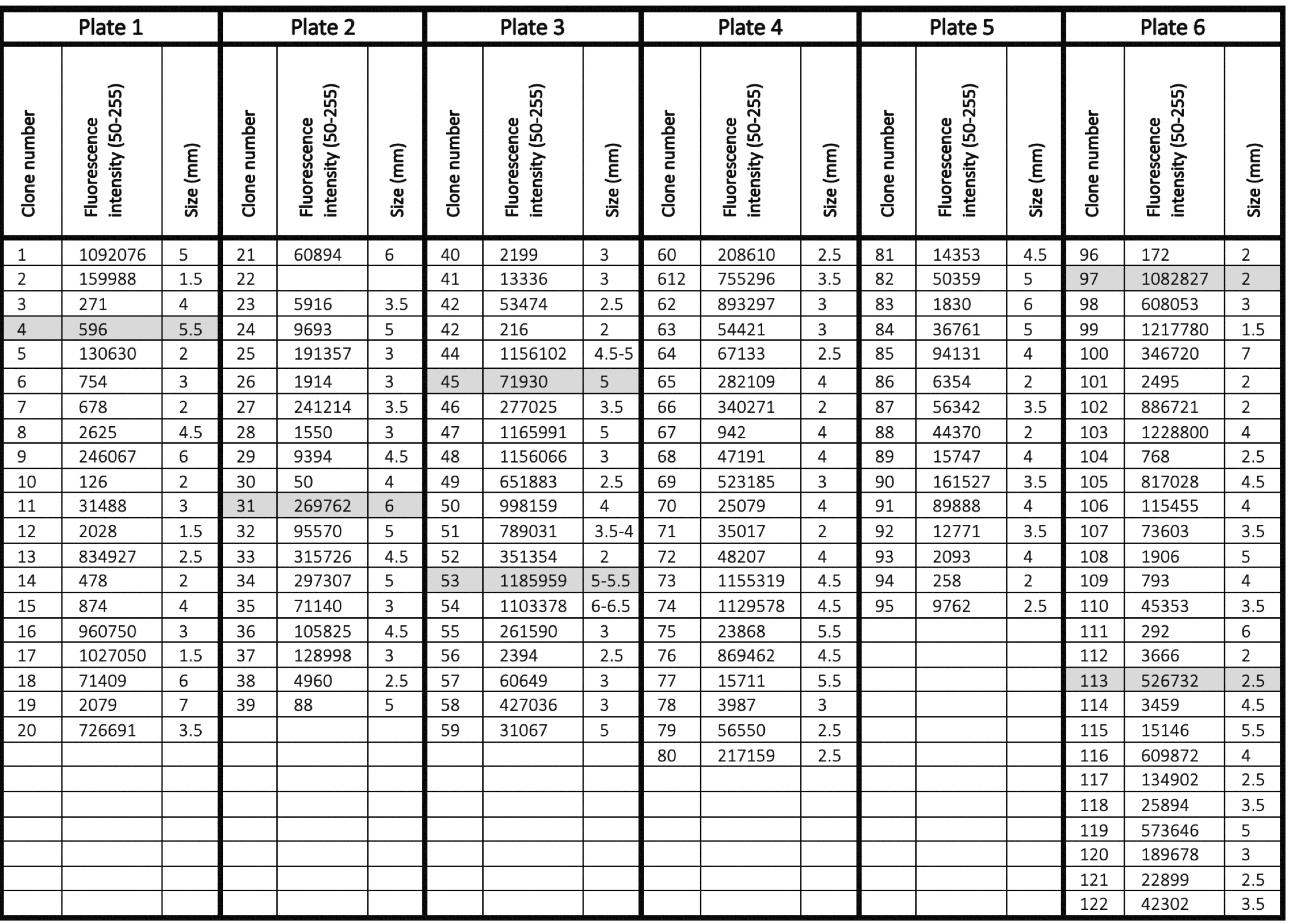


**Supplementary Figure S2**

**
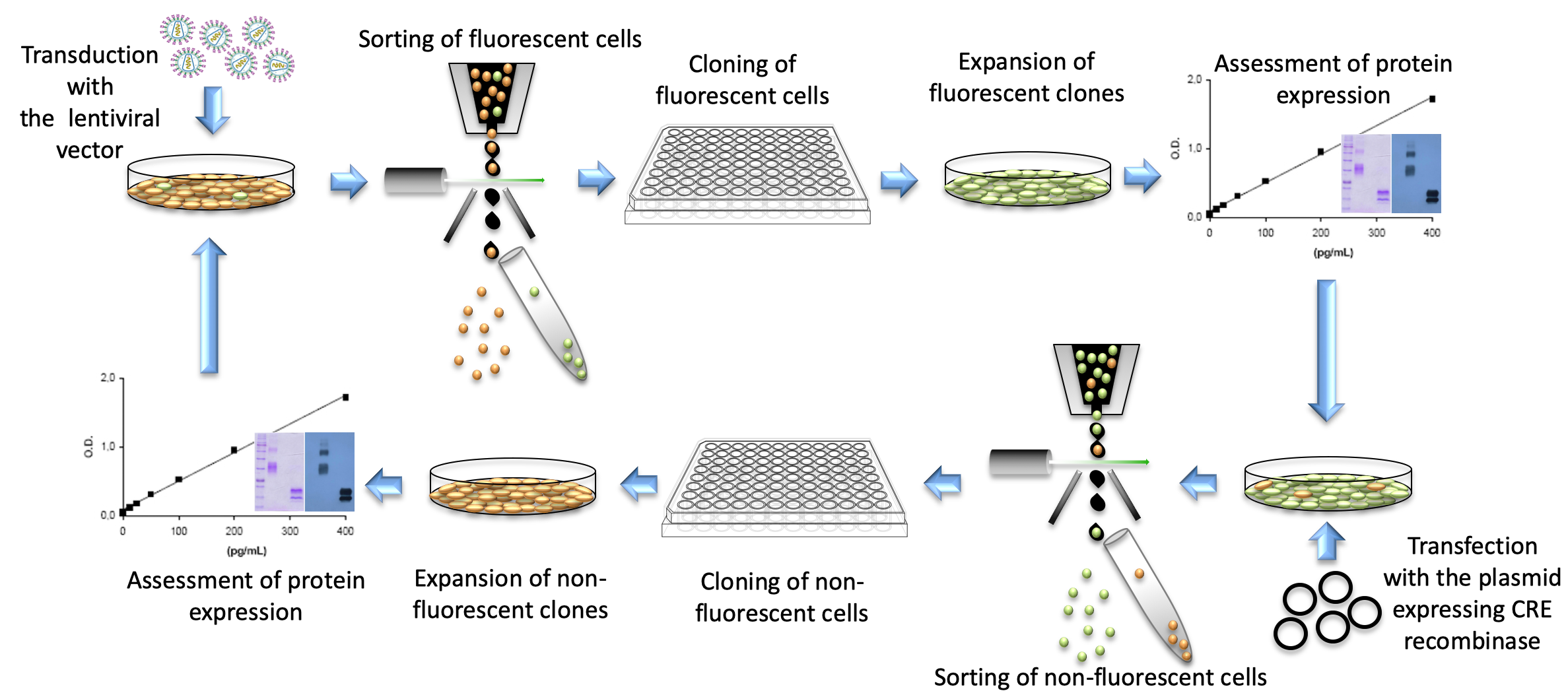
**

**Supplementary fig. S2:** Scheme of fluorescence-assisted sequential insertion of transgenes for generating high-producing clones. This approach requires lentiviral vectors as vehicles for gene transfer. Proviral genomes are usually integrated into transcriptionally active areas avoiding tandem arrays. Within the proviral sequence, the gene of interest and the fluorescence marker (Ex. GFP) are transcribed from a single strong promoter generating a bicistronic mRNA, which guarantees that clones with higher levels of fluorescence also express higher levels of the gene of interest. After transduction with the lentiviral vector, highly fluorescent cells are sorted, amplified, and analyzed for corroborating the expression of the gene of interest. The clone showing the highest expression levels will be transfected with a plasmid encoding a site-specific recombinase (Ex. CRE recombinase). This recombinase removes the fluorescence marker without affecting the gene of interest. Again, the non-fluorescent cells are isolated by FACS, amplified, and analyzed for corroborating the expression of the gene of interest. Finally, these non-fluorescent clones can be subjected to a new round of lentiviral transduction. The entire process can be repeated several times with the aim of increasing step by step the specific productivity.
